# ECG-based gating in ultra high field cardiovascular magnetic resonance using an independent component analysis approach

**DOI:** 10.1186/1532-429X-15-104

**Published:** 2013-11-19

**Authors:** Johannes W Krug, Georg Rose, Gari D Clifford, Julien Oster

**Affiliations:** 1 Department of Electrical Engineering and Information Technology, Otto-von-Guericke University, Magdeburg, Germany; 2 Department of Engineering Science, University of Oxford, Oxford, UK

**Keywords:** Cardiovascular magnetic resonance, CMR, ECG, Gating, Independent component analysis, MHD, VCG

## Abstract

**Background:**

In Cardiovascular Magnetic Resonance (CMR), the synchronization of image acquisition with heart motion is performed in clinical practice by processing the electrocardiogram (ECG). The ECG-based synchronization is well established for MR scanners with magnetic fields up to 3 T. However, this technique is prone to errors in ultra high field environments, e.g. in 7 T MR scanners as used in research applications. The high magnetic fields cause severe magnetohydrodynamic (MHD) effects which disturb the ECG signal. Image synchronization is thus less reliable and yields artefacts in CMR images.

**Methods:**

A strategy based on Independent Component Analysis (ICA) was pursued in this work to enhance the ECG contribution and attenuate the MHD effect. ICA was applied to 12-lead ECG signals recorded inside a 7 T MR scanner. An automatic source identification procedure was proposed to identify an independent component (IC) dominated by the ECG signal. The identified IC was then used for detecting the R-peaks. The presented ICA-based method was compared to other R-peak detection methods using *1)* the raw ECG signal, *2)* the raw vectorcardiogram (VCG), *3)* the state-of-the-art gating technique based on the VCG, *4)* an updated version of the VCG-based approach and *5)* the ICA of the VCG.

**Results:**

ECG signals from eight volunteers were recorded inside the MR scanner. Recordings with an overall length of 87 min accounting for 5457 QRS complexes were available for the analysis. The records were divided into a training and a test dataset. In terms of R-peak detection within the test dataset, the proposed ICA-based algorithm achieved a detection performance with an average sensitivity (*Se*) of 99.2%, a positive predictive value (+*P*) of 99.1%, with an average trigger delay and jitter of 5.8 ms and 5.0 ms, respectively. Long term stability of the demixing matrix was shown based on two measurements of the same subject, each being separated by one year, whereas an averaged detection performance of *Se* = 99.4% and +*P* = 99.7% was achieved.

Compared to the state-of-the-art VCG-based gating technique at 7 T, the proposed method increased the sensitivity and positive predictive value within the test dataset by 27.1% and 42.7%, respectively.

**Conclusions:**

The presented ICA-based method allows the estimation and identification of an IC dominated by the ECG signal. R-peak detection based on this IC outperforms the state-of-the-art VCG-based technique in a 7 T MR scanner environment.

## Background

Cardiac applications have a steadily gaining popularity in Magnetic Resonance Imaging (MRI) [1]. Nowadays, Cardiovascular Magnetic Resonance (CMR) is applied in clinical routine in MR scanners with field strengths up to 3 T. Ultra high field (UHF) (i.e. MRI scanners with 7 T or 9.4 T) CMR applications are becoming more and more popular in the research community [2,3].

Challenges in CMR are the synchronization of the cardiac activity with the acquisition of the MR images and the compensation of motion artefacts during the cardiac cycle [4]. The synchronization is called gating and can be performed prospectively and retrospectively. The state-of-the-art methods are based on the electrocardiogram (ECG) signal [5,6]. These methods rely on the detection of the R-wave, which is a feature more or less easily extracted and occurring while the ventricle cells are depolarized. Other techniques for synchronisation are based on Doppler ultrasound [7], finger plethysmography, self gating techniques [8] or a recently developed phonocardiogram technique [9].

**Figure 1:**
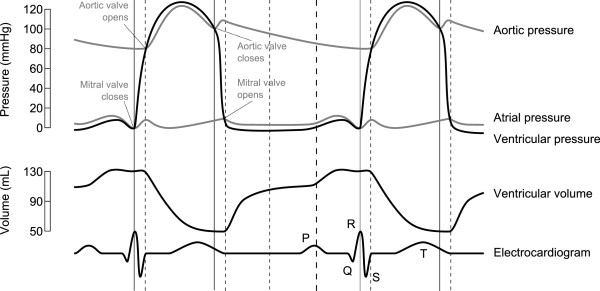
**Wiggers diagram.** The diagram shows the aortic, atrial and ventricular pressure and the ventricular volume in relation to the ECG signal. (Image source: *Wikimedia Commons*^a^), modified).

Inside the MR scanner, ECG signals are severely affected by the magnetohydrodynamic (MHD) effect. The MHD effect results from the interaction between the pulsatile blood flow – which is caused by the rhythmic action of the heart – and the static magnetic field of the MR scanner, **B**_0_[10]. The MHD effect leads to an induction of a voltage across blood vessels which are perpendicular to the MR scanner’s static magnetic field. The resulting body surface potentials of the MHD effect superimpose the ECG signal which exacerbates the detection of the R-peaks. It is assumed that the majority of the MHD-related distortions are caused by the flow of blood in the aortic arch [11,12]. The MHD effect is highest during the ejection phase, i.e. during the ventricular systole, where the blood from the ventricles flows into the aorta and the pulmonary arteries (Figure 1)^a^.

ECG-based gating methods rely on a spatial representation of the heart’s electrical activity: the vectorcardiogram (VCG). These methods assume that the R-wave can be spatially discriminated from the MRI induced artefacts and more specifically from the magnetohydrodynamic (MHD) effect [5,6,13].

However, the VCG-based gating approaches are prone to errors in MR scanners with magnetic field strengths of 7 T and above due to the increased MHD effect at these fields strengths [9,14,15]. In such cases the image acquisition is based on false detections, i.e. missed R-waves and the detection of false positives can occur. Consequently, the resulting MR image is contaminated with motion artefacts and blurring effects.

This work investigates the use of Independent Component Analysis (ICA) for the enhancement of the ECG contribution over the MHD effect in order to improve the R-peak detection and hence the gating ability of the ECG in UHF MRI scanners resulting in an increase of the intrinsic MR image quality. ICA is a statistical technique applied to multidimensional data in order to blindly separate the underlying signal components based on a cost function which maximizes the statistical independence of the resulting components [16,17]. In ECG signal processing, ICA was used for many applications such as the extraction of the foetal ECG, for artefact removal or for the extraction of atrial activity [18-20]. In CMR, ICA was used to reduce the gradient artefacts from 3-lead ECG signals [21]. In this work, ICA is applied to 12-lead ECG signals recorded inside a 7 T MR scanner. The independent component (IC) which is dominated by the ECG signal is automatically identified by a simple classification rule based on a template matching method. The identified IC is used for QRS detection.

## Methods

### ECG database acquisition

**Figure 2:**
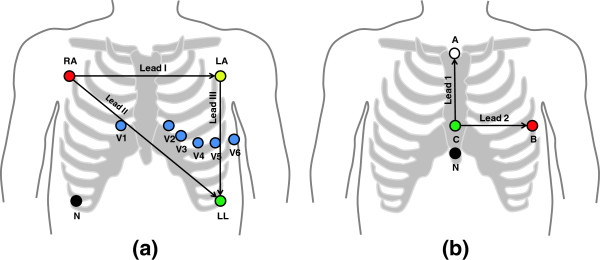
**ECG electrode positioning.** The 12-lead configuration was used for the experimental measurements **(a)**. Typical CMR gating applications employ a reduced orthogonal 2-lead configuration **(b)**.

A standard 12-lead Holter ECG (CardioMem CM3000-12, GETEMED, Germany) with a sampling rate of 1024 Hz, a resolution of 12 bits, an input voltage range of ±6 mV and an analogue bandwidth ranging from 0.05 Hz to 100 Hz was used for recording the ECGs. The recorded ECGs consisted of the limb leads (I, II, III), of the augmented limb leads (aVR, aVL, aVF) and of the six precordial leads (V1-V6). Figure 2(a) shows the electrode positions of a 12-lead ECG as it was used during the experiments. In contrast, Figure 2(b) depicts an orthogonal 2-lead ECG configuration as it is typically used in CMR.

The ECG signals referred to in this work were recorded in a 7.0 T MR scanner (Magnetom, Siemens, Germany). MR imaging was switched off during all ECG measurements and hence gradient and radiofrequency magnetic fields did not exist. The ECG device was placed outside the MR scanner room using lead cable extensions. Standard MR-safe ECG Electrodes were used (H34SG, Covidien, Ireland). The study was approved by the local ethics committee, and written informed consent was obtained from all subjects prior to the ECG measurements. The ECG signals were recorded from eight healthy volunteers (seven male, one female) aged between 24 and 31 years and were recorded outside and inside the MR scanner during normal breathing in supine position. Inside the MR scanner, the ECG measurements were made in a Feet first (Ff) and in a Head first (Hf) position.

A change between the Ff and Hf position inverts the orientation of the MR scanner’s static magnetic field ${\vec{B}}_{0}$ with respect to the volunteer. Hence, the polarity of the MHD effect changes [10,22]. The thorax was positioned in the centre of the MR scanner’s bore during the measurements. The ECG signals were acquired for 2-3 min outside and 4-5 min for each position inside the scanner. Nine different ECG datasets D_1_- D_9_ were recorded inside the MR scanner. Each of the nine datasets consisted of two subsets: one subset containing the Ff (D_1_(Ff) - D_9_(Ff)) and one subset containing the Hf (D_1_(Hf) - D_9_(Hf)) measurements. For the development and evaluation of the ICA-based gating method, the ECG records were separated into a training and a test dataset. D_1_- D_5_ were used as training datasets and D_6_- D_9_ were used as test datasets. Hence, ten ECG records were available from the training dataset (D_1_(Ff)- D_5_(Ff) and D_1_(Hf)- D_5_(Hf)) and eight records were available from the test dataset (D_6_(Ff)- D_9_(Ff) and D_6_(Hf)- D_9_(Hf)). The ten subsets of D_1_- D_5_ (training dataset) had a total length of 47 min corresponding to 2853 R-peaks. The eight subsets of D_6_- D_9_ (test dataset) had a total length of 40 min corresponding to 2604 R-peaks. Test dataset D_9_ was recorded from the same volunteer as the training dataset D_5_, but D_5_ was recorded one year prior to D_9_.

**Figure 3:**
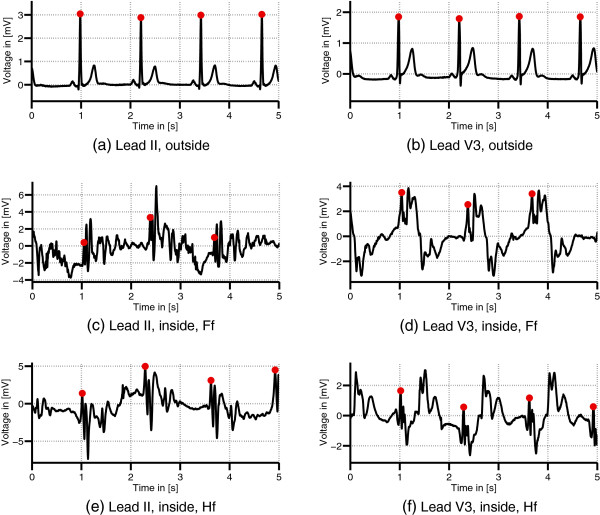
**ECGs acquired outside and inside the MRI.** Comparison of ECG leads II and V3 of dataset D_1_ acquired outside **(a)-(b)** and inside the 7 T MR scanner in Ff **(c)-(d)** and Hf position **(e)-(f)**. The dots mark the positions of the R-peaks.

**Figure 4:**
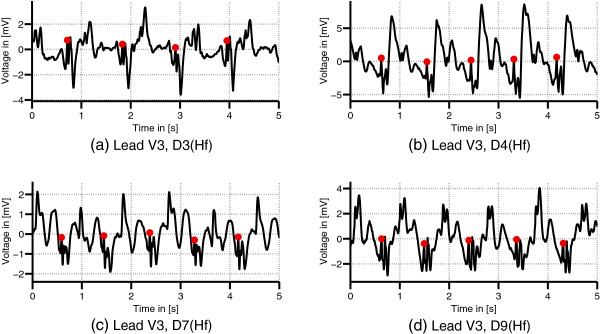
**Variation of the MHD effect.** ECG records (lead V3) from different volunteers acquired in the head first (Hf) position **(a)-(d)**. The MHD effect varies between the different datasets. The dots mark the positions of the R-peaks.

Additional ECG datasets recorded outside the MR scanner from each volunteer were used for the generation of QRS templates, for comparing different signal properties and for the application of the VCG-based algorithms. Figure 3(a)- 3(f) show ECG signals of leads II and V3 measured outside and inside the 7 T MR scanner. Figure 4 depicts the variations of the MHD effect of the precordial lead V3 in four different datasets recorded in the Hf position.

### ICA-based suppression of the MHD effect

The ECG signals recorded inside the MRI scanner were contaminated by the MHD and other noise components. This linear mixture of the different signal components can be described by

(1) $$\begin{array}{l} x_{k}={\text{As}}_{k} \end{array}$$

where **A** is the so-called (linear) mixing matrix, **s**_*k*_ is a vector containing the source signals and **x**_*k*_ is the measurement signal vector containing the mixture of the source signals at time instant *k*. In order to separate the ECG from the MHD and other noise components, an estimate of the real source signals, ${\hat{\text{s}}}_{k}$, was required. Therefore, ICA was applied to the measured data vector **x**_*k*_ to find a demixing matrix **W** so that:

(2) $${\hat{\text{s}}}_{k}={\text{Wx}}_{k}$$

where ${\hat{\text{s}}}_{k}$ are the estimated source signals or independent components (ICs). Several algorithms have been proposed to solve this problem which differ mainly from the cost function used for measuring the Gaussianity (which is a measure of independence) of the signals. In this work the FastICA algorithm was used [23]. The FastICA algorithm was not adapted to the specific problem discussed in this work. Where required, the dimensionality of **x**_*k*_ was reduced by limiting the number of eigenvalues or principal components (PCs) prior to the application of ICA.

From the estimated ICs ${\hat{\text{s}}}_{k}$, one IC ${\hat{s}}_{\text{k,ECG}}$ which was dominated by the R-peak or QRS complex needed to be identified. This pivotal step is described in the following section.

### Identification of an independent component for gating

One crucial step with the application of ICA to the given problem was the identification of the IC ${\hat{s}}_{\text{k,ECG}}$. This IC was required to represent the cardiac activity, i.e. it should contain the R-peak so that standard R-peak/QRS detection algorithms could be applied. For the practical application of the proposed method, an automated identification of $\;{\hat{s}}_{\text{k,ECG}}$ was required.

**Figure 5:**
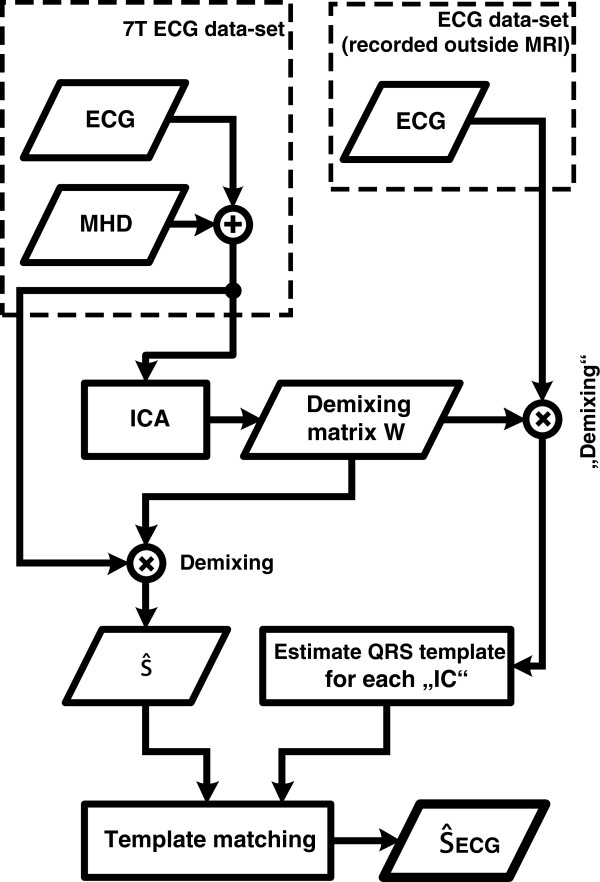
**Automated procedure for the identification of** ${\hat{s}}_{\text{k,ECG}}$**.** A template matching algorithm was employed for the identification of ${\hat{s}}_{\text{k,ECG}}$. The demixing matrix **W** obtained from the ECG signals acquired inside the MR scanner (**x**_*k*_) was applied to the ECG signals acquired outside the MR scanner (**x**_*k*,*out*_). A QRS template was generated from each IC. ${\hat{s}}_{\text{k,ECG}}$ was identified by cross-correlating each QRS template with the corresponding IC in ${\hat{\text{s}}}_{k}$.

A template matching algorithm depicted in Figure 5 was used for an automated identification of $\;{\hat{s}}_{\text{k,ECG}}$. The demixing matrix **W** which was estimated from the disturbed ECG signal **x**_*k*_ was applied to the ECG **x**_*k*,*out*_ acquired outside the MR scanner resulting in a set of linear combinations $\;{\hat{\text{s}}}_{\text{k,out}}={\mathrm{Wx}}_{k,\text{out}}$. For each of these linear combinations, a template **u**_k,qrs_ of the QRS complex was generated using the following procedure. First, a QRS detection algorithm [24] was applied to the clean ECG signal in order to estimate the positions of the QRS complexes. The QRS template for each linear lead combination ${\hat{\text{s}}}_{\text{k,out}}$ was then estimated from segments of 80 ms length centred around the detected QRS positions and averaged over ten consecutive QRS complexes. For the identification of ${\hat{s}}_{\text{k,ECG}}$, the cross-correlation functions $\left({\hat{\text{s}}}_{k}⋆{\text{u}}_{\text{k,qrs}}\right)$ between each QRS template in **u**_k,qrs_ and the corresponding IC in ${\hat{\text{s}}}_{k}$ were calculated. Based on the maximum value of the cross-correlation functions, one IC in ${\hat{\text{s}}}_{k}$ was identified as ${\hat{s}}_{\text{k,ECG}}$. The IC corresponding to ${\hat{s}}_{\text{k,ECG}}$ in ${\hat{\text{s}}}_{\text{k,out}}$ is referred to as ${\hat{s}}_{\text{k,out,ECG}}$.

### Description of the proposed method

**Figure 6:**
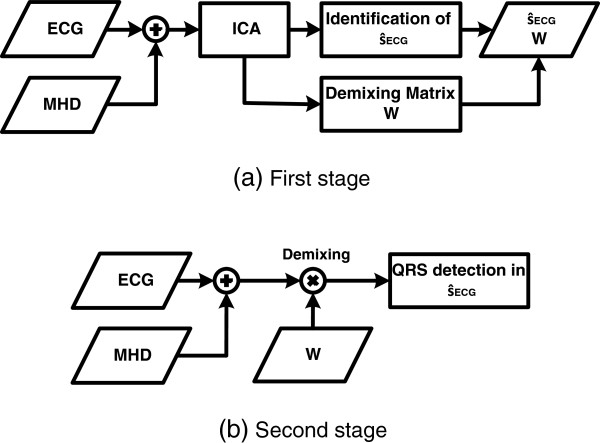
**Estimation of the demixing matrix W and its application.** **(a)**: Using a 30 s long ECG signal, the demixing matrix **W** and the IC ${\hat{s}}_{\text{k,ECG}}$ were estimated. **(b)**: The demixing matrix **W** obtained in the first stage was applied the the latest ECG sample. This procedure was followed by applying an QRS detector to ${\hat{s}}_{\text{k,ECG}}$.

The extraction of ${\hat{s}}_{\text{k,ECG}}$ from the initial ECG signals and the subsequent R-peak detection were applied in a two-stage process (Figure 6).

In the *first stage*, ICA was applied to a 30 s reference segment **x**_Ref,k_ of the 12-lead ECG signal which was recorded inside the MR scanner. The resulting demixing matrix **W** was used to transform the reference vector **x**_Ref,k_ into the vector containing the estimated sources, i.e. ${\hat{\text{s}}}_{k}$. This is described by Eq. 2. The estimated signals contained in ${\hat{\text{s}}}_{k}$ were dominated by different (physiological) signal sources, e.g. by the ECG signal itself, by the MHD effect, by respiratory motion artefacts or by measurement noise. After the identification of ${\hat{s}}_{\text{k,ECG}}$, the demixing matrix **W** was reduced to a demixing vector **w** since only ${\hat{s}}_{\text{k,ECG}}$ was required for the R-peak detection. The estimation of ${\hat{s}}_{\text{k,ECG}}$ can be summarized as the scalar product of **w** and **x**_*k*_:

(3) $${\hat{s}}_{\text{k,ECG}}=w\cdot x_{k}$$

where **w** is a row vector and **x**_*k*_ is a column vector with each row in **x**_*k*_ corresponding to one of the twelve ECG leads at time instant *k*. The estimated demixing vector **w** remained unchanged, i.e. it was only estimated once for each dataset.

In the *second stage*, the demixing vector **w** was applied to the most recent ECG sample **x**_*k*_ resulting in a real-time estimation of ${\hat{s}}_{\text{k,ECG}}$. The demixing process was followed by the application of a R-peak/QRS detection algorithm to ${\hat{s}}_{\text{k,ECG}}$[24].

This R-peak detection algorithm uses the first derivative of the ECG signal to enhance the QRS complex. To ensure a reliable R-peak detection inside the MR scanner, the slope of the QRS complex has to be preserved in ${\hat{s}}_{\text{k,ECG}}$. This was investigated quantitatively by comparing the upslope differences of the QRS complex in the identified IC ${\hat{s}}_{\text{k,ECG}}$ and in the corresponding IC ${\hat{s}}_{\text{k,out,ECG}}$. After testing both distributions (upslopes in ${\hat{s}}_{\text{k,ECG}}$ and in ${\hat{s}}_{\text{k,out,ECG}}$) for normality using a Lilliefors test [25], a two-sample *t*-test (*α* = 0.05) was conducted to test if the mean slopes of ${\hat{s}}_{\text{k,ECG}}$ and ${\hat{s}}_{\text{k,out,ECG}}$ showed significant differences.

### ECG lead configurations used for estimating ${\hat{\text{s}}}_{k}$

For the estimation of ${\hat{\text{s}}}_{k}$, ICA was applied to different combinations of the recorded ECG leads. The aim of this experiment was to investigate how the additional precordial ECG leads affected the quality of the R-peak detection results.

At the first step, ICA was applied to 8 of the 12 leads contained in each ECG dataset. These were the limb leads I and II and the precordial leads V1-V6. The remaining 4 leads (III, aVR, aVL and aVF) were excluded because they can be derived from linear combinations of limb leads I and II. Hence, these 4 leads contained no additional information. The usage of 8 leads for the estimation of ${\hat{\text{s}}}_{k}$ is referred to as lead configuration LC1.

In the second step, ICA was applied to the same datasets but fewer ECG leads were used. These datasets consisted of combinations of the limb leads I and II and of different precordial leads. Furthermore, the influence of decreasing the dimensionality of the datasets was investigated. This was achieved by a reduction of the principal components (PCs) before ICA was applied to the datasets. These combinations of varying ECG leads and PCs are referred to as lead configurations LC2a-LC2h. Results for methods LC2 using leads I, II and at least two precordial lead are given for the best lead/PC combinations. Results for different combinations of lead I and II and of one precordial lead are computed to highlight the influence of a single precordial lead on the R-peak detection (LC2e-LC2g). Finally, ICA was applied to lead I and II without any additional precordial lead (LC2h).

### Other R-peak detection methods (M1-M5)

Besides the ICA-based method for QRS detection, other methods for detecting the R-peaks in the contaminated datasets were investigated. The five other R-peak detection techniques, called methods M1-M5, were applied to the 12-lead ECG signals which were acquired using the electrode configuration shown in Figure 2(a). The 3D VCG **v**(*k*) = (*v*_*x*_(*k*),*v*_*y*_(*k*),*v*_*z*_(*k*)) required for methods M2-M5 was obtained from the 12-lead ECG signals using the inverse Dower matrix [26]. An overview of the five different methods M1-M5 which were implemented for comparison with the proposed ICA-based method is given below. Methods M1, M2 and M5 used the R-peak detector from [24]. R-peak detection results using methods M1-M5 are given for the training and test dataset.

#### R-peak detection in a single ECG lead (M1)

The QRS detection algorithm was successively applied to each of the 12 leads in all datasets. Finally, one lead was chosen which gave the best results averaged over all datasets. Results are given for this single lead.

#### R-peak detection in a single VCG lead (M2)

QRS detection was performed in all three VCG leads **v**(*k*) = (*v*_*x*_(*k*),*v*_*y*_(*k*),*v*_*z*_(*k*)). Results are presented for the same lead in all datasets which gave the best average detection results.

#### 3D VCG-based R-peak detection (M3)

The VCG-based gating method was implemented according to [5,13]. A reference vector **r**_out_ = (*r*_*x*_,*r*_*y*_,*r*_*z*_) which points at the spatial position of the R-peak was obtained for each dataset based on the clean ECG signal acquired outside the MR scanner. The distance and angle between **r**_out_ and the VCG vector **v**(*k*) was calculated. Those positions of the vector **v**(*k*) where the distance and angle between **r**_out_ and **v**(*k*) fell below a threshold were defined as R-peaks.

#### Modified 3D VCG-based R-peak detection (M4)

As explained in the introduction, the R-peak detection based on the 3D VCG (method M3) is prone to errors in 7 T MR scanners due to the altered amplitude and angle of the R-peak vector in the actual VCG **v**(*k*). To cope with this problem, a modified version of the VCG-based method was implemented. This method calculated a reference vector **r**_in_ from the ECG signal which was recorded inside the MR scanner instead of obtaining it from the ECG recorded outside the scanner as described by method M3. The remaining procedure of the VCG-based algorithm remained unchanged.

#### ICA of the VCG for R-peak detection (M5)

To further analyse the usability of the VCG signal **v**(*k*), ICA was applied to the three VCG leads *v*_*x*_(*k*),*v*_*y*_(*k*) and *v*_*z*_(*k*) of each dataset. As for the ICA-based method, one IC ${\hat{s}}_{\text{k,ECG}}$ was identified using the template matching process.

### Evaluation of the different gating methods

#### R-peak detection performance

The QRS detection performance using the different methods was evaluated by the sensitivity (*Se*) and the positive predictive value (+*P*):

(4) $$\begin{array}{ll} \;\text{Se} & =\frac{\text{TP}}{\text{TP}+\text{FN}}\\ \;+P & =\frac{\text{TP}}{\text{TP}+\text{FP}} \end{array}$$

where *TP* is the number of true positives, *FP* the number false positives and *FN* the number of false negatives. These parameters were estimated according to the ANSI/AAMI EC57 standard recommendations [27]. A manual annotation of the R-peaks was used as gold standard for performance estimation of the different R-peak detection approaches. The manual annotations were made by two ECG experts.

#### Mean trigger propagation delay *μ*_pd_ and jitter *σ*_pd_

The stability of the trigger timing was evaluated based on the propagation delay and the jitter. The mean trigger propagation delay *μ*_pd_ was defined as the difference between the position of the manually annotated R-peak and the R-peak detected by the QRS detection algorithm and was averaged over each dataset.

#### Long term stability and robustness of the proposed ICA-based gating method

In order to calculate the demixing matrix **W** only once per volunteer at the beginning of the measurement, the long term stability of **W** had to be investigated. The training set D_5_ and the test set D_9_ – which were acquired from the same volunteer but were separated by one year – were used for this experiment. Two demixing matrices ${\text{W}}_{{\text{D}}_{5}(\text{Ff})}$ and ${\text{W}}_{{\text{D}}_{5}(\text{Hf})}$ obtained from the datasets D_5_(Ff) and D_5_(Hf) were applied to the two corresponding datasets D_9_(Ff) and D_9_(Hf) resulting in two ICs ( ${\hat{s}}_{\text{k,ECG}}$) which were used for R-peak detection. R-peak detection results are given in terms of *Se* and +*P* for both datasets.

## Results

### ICA-based approach

#### Estimation of ${\hat{s}}_{k}$, identification of ${\hat{s}}_{k,\text{ECG}}$ and slope of the QRS complex in ${\hat{s}}_{k,\text{ECG}}$ and ${\hat{s}}_{k,\text{out}}$

**Figure 7:**
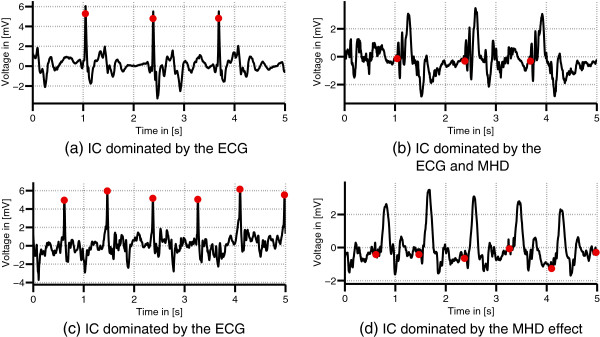
**Exemplary ICs.** Different ICs obtained from datasets D_1_(Ff) **(a)-(b)** and D_2_(Ff) **(c)-(d)**. The ICs ${\hat{s}}_{\text{k,ECG}}$ shown in **(a)** and **(c)** were used for R-peak detection.

Four exemplary ICs from two different datasets obtained by FastICA are shown in Figure 7: two ICs mainly dominated by the ECG, one dominated by the ECG and the MHD effect and one IC mainly dominated by the MHD effect. The IC dominated by the MHD effect is characterized by an attenuation of the R-peak’s amplitude. None of the ICs could be clearly assigned to a certain signal source since each IC contained a mixture of ECG, MHD and noise components.

The IC ${\hat{s}}_{\text{k,ECG}}$ required for R-peak detection was identified in all datasets based on the template matching process described in the methods section.

**Figure 8:**
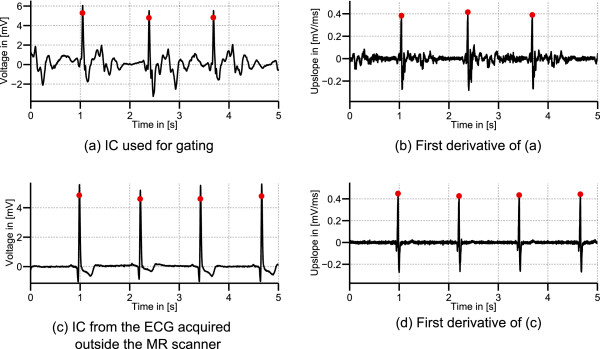
**IC used for gating, its first derivative, IC from the ECG acquired outside the MR scanner and first derivative.** IC from dataset D_1_(Ff) identified as ${\hat{s}}_{\text{k,ECG}}$**(a)** and its first derivative with detected R-peaks positions **(b)**. **(c)**: ${\hat{s}}_{\text{k,out,ECG}}$ obtained by applying the demixing matrix **W** to the ECG acquired outside the MR scanner. **(d)**: The first derivative of **(c)**.

**Figure 9:**
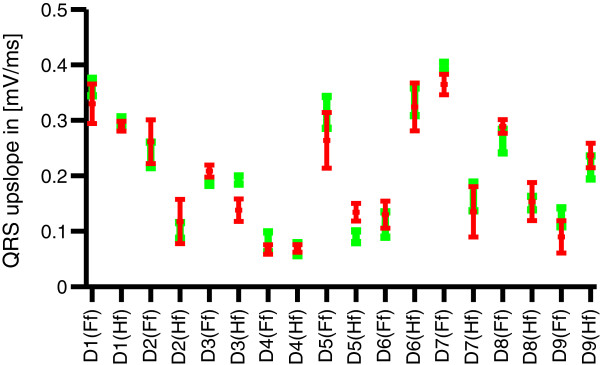
**QRS upslopes.** Mean and standard deviations of the QRS-upslopes for all datasets in ${\hat{s}}_{\text{k,ECG}}$ (red) and of the corresponding IC ${\hat{s}}_{\text{k,out,ECG}}$ (green).

Figure 8(a)-(b) depict the IC ${\hat{s}}_{\text{k,ECG}}$ used for gating and its first derivative. In Figure 8(c), the IC ${\hat{s}}_{\text{k,out,ECG}}$ obtained from the ECG acquired outside the MR scanner is shown. The first derivative or upslope of ${\hat{s}}_{\text{k,out,ECG}}$ is depicted in Figure 8(d). Using the Lillifors test it was shown that the upslopes of the QRS complex measured in ${\hat{s}}_{\text{k,ECG}}$ and ${\hat{s}}_{\text{k,out,ECG}}$ exhibit a normal distribution [25]. Figure 9 compares the mean and standard deviations of the QRS-upslopes in ${\hat{s}}_{\text{k,ECG}}$ to those of the corresponding ICs ${\hat{s}}_{\text{k,out,ECG}}$. The average absolute relative deviations of the slopes in ${\hat{s}}_{\text{k,ECG}}$ compared to ${\hat{s}}_{\text{k,out,ECG}}$ were 14.1% in the training set and 16.7% in the test set. The two-sample t-test revealed a significant change of the slope’s mean value.

#### R-peak detection in ${\hat{s}}_{\text{k,ECG}}$ using lead configurations LC1 and LC2

Detailed results for the R-peak detection in ${\hat{s}}_{\text{k,ECG}}$ using lead configuration LC1 are given in Table 1 for the training dataset and in Table 2 for the test dataset. For the training dataset, an average sensitivity *Se* of 99.5% and an average positive predictive value +*P* of 99.4% were achieved. The average values of the mean propagation delay *μ*_pd_ and jitter *σ*_pd_ were 3.5 ms and 6.5 ms, respectively. Dataset D_4_(Ff) shows a higher jitter when compared to the other datasets. Comparable R-peak detection results were achieved for the test dataset with *Se* = 99.2%, +*P* = 99.1%, *μ*_pd_ = 5.8 ms and *σ*_pd_ = 5.0 ms. Exemplary sections of R-peaks in ${\hat{s}}_{\text{ECG}}$ are shown in Figures 7(a), 7(c) and 8(a).

**Table 1:** ICA-based R-peak detection results for the training dataset

**Dataset**	**# of R-peaks**	***Se* [%]**	**+** ***P* [%]**	***μ***_**pd**_** [ms]**	***σ***_**pd**_** [ms]**
D_1_(Ff)	165	100.0	100.0	4.1	4.4
D_1_(Hf)	226	100.0	98.7	1.6	0.5
D_2_(Ff)	285	99.6	99.6	1.1	3.3
D_2_(Hf)	198	97.5	100.0	4.1	2.8
D_3_(Ff)	243	98.4	99.6	6.4	10.6
D_3_(Hf)	254	100.0	99.6	1.7	4.1
D_4_(Ff)	361	100.0	100.0	0.5	18.2
D_4_(Hf)	400	99.8	100.0	4.5	0.6
D_5_(Ff)	326	100.0	99.7	8	11.3
D_5_(Hf)	395	99.5	96.3	4.1	9.9
Total	2853	-	-	-	-
Mean	-	99.5	99.4	3.5	6.5

Detailed R-peak detection results for the ten ECG records of the training dataset using the ICA-based gating method with lead configuration LC1 (*μ*_pd_: mean detection delay; *σ*_pd_: jitter).

**Table 2:** ICA-based R-peak detection results for the test dataset

**Dataset**	**# of R-peaks**	***Se* [%]**	**+** ***P* [%]**	***μ***_**pd**_** [ms]**	***σ***_**pd**_** [ms]**
D_6_(Ff)	401	100	99.5	13.4	9.6
D_6_(Hf)	416	98.6	99.8	2.2	4.8
D_7_(Ff)	202	98	97.9	3.6	0.8
D_7_(Hf)	163	100	97.6	1.9	7
D_8_(Ff)	310	100	100	0.3	0.5
D_8_(Hf)	421	98.8	99.5	1.1	0.7
D_9_(Ff)	331	99.7	99.1	11.1	9.3
D_9_(Hf)	360	98.1	99.7	12.9	7.6
Total	2604				
Mean		99.2	99.1	5.8	5.0

Detailed R-peak detection results for the eight ECG records of the test dataset using the ICA-based gating method with lead configuration LC1 (*μ*_pd_: mean detection delay; *σ*_pd_: jitter).

Table 3 summarizes the R-peak detection results in ${\hat{s}}_{\text{k,ECG}}$ using lead configurations LC2a-LC2h. *Se* and +*P* slightly decreased when less precordial leads were used. Compared to lead configuration LC1, the mean propagation delay *μ*_pd_ and jitter *σ*_pd_ increased. The results for the 3-lead configurations LC2e-LC2g revealed the impact of the precordial leads V2-V4 on the overall R-peak detection quality. Out of these 3-lead configurations, LC2g using the precordial lead V4 gave the best R-peak detection results (*Se* = 98.2%, +*P* = 97.3% for the training dataset and *Se* = 98.5%, +*P* = 97.2% for the test dataset). Without using any of the precordial leads but only limb leads I and II as in configuration LC2h, *Se* and +*P* decreased to 77.1% and 66.3% for the training dataset and to 78.9% and 65.8% for the test dataset.

**Table 3:** ICA-based R-peak detection results for the training and test datasets using different lead configurations

	**Configuration**			**Training dataset**				**Test dataset**			
**LC**	**# leads**	**# PCs**	**Leads I, II +**	***Se* [%]**	**+** ***P* [%]**	***μ***_**pd**_** [ms]**	***σ***_**pd**_** [ms]**	***Se* [%]**	**+** ***P* [%]**	***μ***_**pd**_** [ms]**	***σ***_**pd**_** [ms]**
1	8	8	V1-V6	99.5	99.4	3.5	6.6	99.2	99.1	5.8	5.0
2a	7	6	V2-V6	99.2	99.1	5.9	9.6	98.9	99	12.1	8.2
2b	6	6	V1-V4	99.2	99.3	4.5	5.6	99.4	98.3	11.7	13.3
2c	5	4	V2-V4	99.2	99.2	4.5	10.3	98.7	97	14.5	13.6
2d	4	4	V3-V4	98.9	98.5	4.5	6.9	98.3	97.7	16.9	13.4
2e	3	3	V2	94.7	94.5	12.2	10.5	95.2	93.7	12.1	15.9
2f	3	3	V3	97.3	92.8	11.7	8.5	96.5	92.4	14.5	9.7
2g	3	3	V4	98.2	97.3	6.3	11.5	98.5	97.2	13.9	12.2
2h	2	2	–	77.1	66.3	33.3	20.6	78.9	65.8	22.1	11.2

R-peak detection results for the training and test datasets using the ICA-based method with lead configurations LC2a-LC2g. Averaged results of lead configuration LC1 are shown for comparison (*μ*_pd_: mean detection delay; *σ*_pd_: jitter).

#### Long term stability of the demixing matrix

Two different ICs ${\hat{s}}_{\text{k,ECG}}$ were obtained after applying the demixing matrices ${\text{W}}_{{\text{D}}_{5}(\text{Ff})}$ and ${\text{W}}_{{\text{D}}_{5}(\text{Hf})}$ to the corresponding ECG datasets D_9_(Ff) and D_9_(Hf) acquired from the same volunteer. Using these two ICs, the following R-peak detection results were achieved: 

• D_9_(Ff): *Se* = 99.7%, +*P* = 99.6%

• D_9_(Hf): *Se* = 99.1%, +*P* = 99.7%

### Other R-peak detection methods

Table 4 compares the results obtained from the gating methods M1-M5 to the results of the ICA-based approach using lead configuration LC1.

**Table 4:** R-peak detection results for methods M1-M5

	**Training dataset**				**Test dataset**			
**Method**	***Se* [%]**	**+** ***P* [%]**	***μ***_**pd**_** [ms]**	***σ***_**pd**_** [ms]**	***Se* [%]**	**+** ***P* [%]**	***μ***_**pd**_** [ms]**	***σ***_**pd**_** [ms]**
ICA LC1	99.5	99.4	3.5	6.5	99.2	99.1	5.8	5.0
M1	86.8	88.4	0.2	2.3	87.1	89.4	23.8	19.1
M2	89.2	96.7	4.1	4.7	88.9	91.2	3.4	2.3
M3	78.3	54.4	7.1	4.4	72.1	57.5	8.9	3.8
M4	79.3	82.3	5.0	5.9	75.6	77.2	6.1	5.4
M5	88.7	86.3	6.9	9.2	84.3	87.5	5.2	10.7

R-peak detection results for methods M1-M5. Results are given for the training and test dataset. Averaged results of the ICA based method with lead configuration LC1 is shown for comparison (*μ*_pd_: mean detection delay; *σ*_pd_: jitter).

The results given for the ECG-based method (M1) were estimated from the precordial lead V4 in all datasets because this ECG lead yielded the best R-peak detection in terms of the average *Se* and +*P*. Whereas the MHD induced contaminations in the limb leads resulted in a lower R-peak detection quality, the precordial leads V3-V5 showed less contaminations and hence better R-peak detection results. Figure 3 depicts this difference between the limb lead II and the precordial lead V3.

The usage of the VCG lead *x* for R-peak detection in method M2 lead to an increased *Se* and +*P* when compared the detection results in method M1.

**Figure 10:**
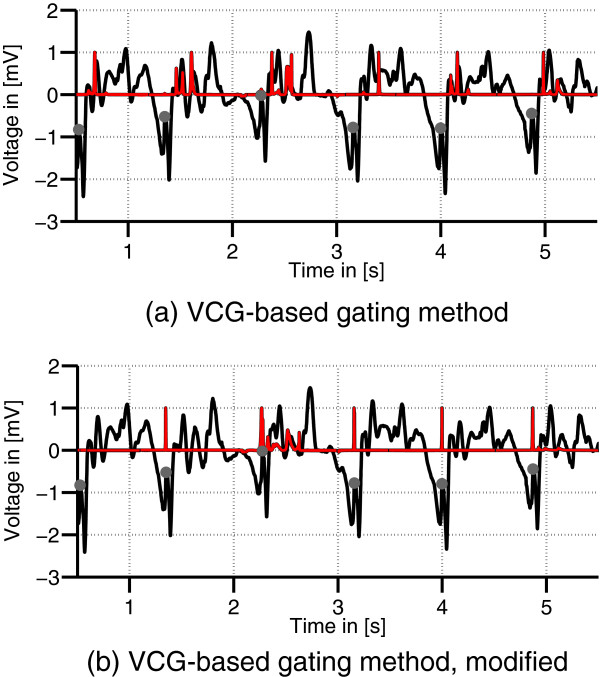
**VCG-based gating results.** VCG lead z (black) of a 7 T record. The dots mark the R-peak positions. The red graph displays the result of the VCG gating algorithm **(a)** and of its modification using the different reference vector **r**_out_ **(b)**.

The state-of-the-art VCG-based gating method (M3) resulted in a *Se* and +*P* within the test dataset of 72.1% and 56.4%, respectively. The usage of a modified reference vector **r**_in_ in method M4 lead to an increase of +*P* of approximately 21%. A comparison of both methods for one dataset is shown in Figure 10. Figure 10(a) depicts the results from the unmodified VCG-based approach (M3). This approach resulted in multiple peaks per R-peak which lead to the low +*P* given in Table 4. In contrast, the results from the VCG-based approach depicted in Figure 10(b) which used a modified reference vector **r**_in_ (M4) showed less peaks per R-peak resulting in a higher +*P*.

The application of ICA to the three VCG leads in method M5 resulted in a decreased *Se* and +*P* when compared to method M2 where QRS detection was directly performed in one of the VCG leads.

## Discussion

### ICA-based gating method

Although it was shown previously that ICA is not suitable for recovering the diagnostic information contained in the MHD-contaminated ECG signal [28], ICA was successfully applied to 12-lead ECG signals for the real-time estimation of an IC ${\hat{s}}_{\text{k,ECG}}$ which was dominated by the R-peak. This IC was then used for R-peak detection. The influence of different ECG leads on the results achieved by ICA was investigated by applying ICA to different lead combinations. The best R-peak detection results were achieved with lead configuration LC1 where eight ECG leads (I,II,V1-V6) were used for the estimation of ${\hat{s}}_{\text{k,ECG}}$. For the combinations of only three ECG leads used with ICA, it was shown that the best average R-peak detection results were achieved by a combination of the two limb leads I and II and of the precordial lead V4. These experiments emphasized the importance of the precordial leads – especially of lead V4 – for the proposed method. Not using the precordial leads as in configuration LC2h caused a substantial decrease in R-peak detection performance (Table 3).

For 7 T CMR application based on a prospective gating scheme, the mean trigger propagation delay *μ*_pd_ should be less than 20 ms because the systole’s mechanical contraction begins 30 ms-70 ms after the R-peak [29]. A large jitter *σ*_pd_ could lead to blurring of the CMR image, to ghost artefacts in the phase encoding direction or to a false estimation of the blood flow rate in phase contrast MRI. For the estimation of blood flow rates, the jitter should be less than 15 ms [30]. Using the proposed ICA-based method with lead configuration LC1, a mean detection delay *μ*_pd_ of 5.8 ms and a jitter *σ*_pd_ of 5.0 ms were achieved in the test dataset using eight ECG leads in lead configuration LC1. Hence, the usage of this method would enable the acquisition of proper CMR images.

The demixing matrix **W** was only estimated once for each dataset using a 30 s segment of the recorded ECG signal and was reduced to a demixing vector **w** after ${\hat{s}}_{\text{k,ECG}}$ was identified. This allowed a demixing of the different signal components and the estimation of ${\hat{s}}_{\text{k,ECG}}$ in real-time. **W** was stable over a long period of time (1 year) which was shown using demixing matrices from previously recorded 7 T datasets of the same volunteer – even though the ECG electrode positions slightly varied between both measurements. Besides, the demixing matrix **W** was obtained from and applied to datasets which were acquired during normal breathing. Large baseline shifts caused by the physical movement of the ECG electrodes during breathing can affect the ECG signals. These variations can complicate the R-peak detection. The results obtained in this study show that the proposed method for the estimation of ${\hat{s}}_{\text{k,ECG}}$ can be applied over a long period of time and is robust against variations caused by normal breathing.

The demixing matrices **W** varied between the different datasets D_1_-D_9_. It was however noticed that the precordial leads were contributing more for the generation of the IC ${\hat{s}}_{\text{k,ECG}}$. Nevertheless, the combination of the precordial leads varied over the subject population, and it was also shown in this study that the limb leads add information and allow for a better separation of the MHD and the ECG. Two reasons can be given for the variation of the demixing matrices. Firstly, the anatomy and physiology of the different volunteers leads to high variations of the MHD signals as it is shown in Figure 4 and was previously discussed in [31]. Hence, the ECG leads were not affected in the same way in the datasets D_1_-D_9_ and different combinations of the measured ECG leads **x**_*k*_ were required to obtain an IC ${\hat{s}}_{\text{k,ECG}}$ which was suitable for R-peak detection. Secondly, this phenomenon can be explained by the positions of the ECG electrodes with respect to the position and orientation of the heart’s electrical axis. These anatomical parameters vary between the different volunteers or datasets. As a result, the ECG traces – either recorded outside or inside the MR scanner – vary between the different volunteers. This requires one initial computation of the demixing matrix **W** for each new subject. In this work it was shown that ICA provides an elegant way of estimating **W** for different subjects without further assumptions.

A similar ICA-based method was utilized previously for the suppression of gradient artefacts from 3-lead ECG signals acquired during MRI [21]. These gradient induced artefacts exhibit temporal variations due to different MRI sequences, slice locations and orientations. Hence, a continuous update of the demixing matrix was required during runtime. As described in this work, an update of the demixing matrix for the suppression of the MHD effect using the 12-lead ECG signals was not necessary.

Given the results discussed above, the presented ICA-based method is suitable to be used in 7 T CMR imaging for the real-time estimation of an IC ${\hat{s}}_{\text{k,ECG}}$ which can then be used for R-peak detection and gating.

### Comparison to other gating methods

From the other R-peak detection techniques M1-M5, method M2 using VCG lead x gave the best results. For method M1 which utilized the original ECG signal, the best R-peak detection results were achieved using the precordial ECG lead V4. Together with the results obtained by the ICA-based method, this again emphasizes the importance of the precordial leads for R-peak detection in 7 T CMR.

The results obtained for the state-of-the-art VCG-based gating method (M3) proposed in [5,13] showed low *Se* and +*P* values which are insufficient for gating at 7 T CMR. Based on the functional principle of the VCG method, a modified reference vector was suggested which increased +*P* within the test dataset by approximately 21%. Although this modification improved the VCG-based technique, results are still not sufficient for a proper gating in CMR. Furthermore, the required manual R-peak annotation makes this modification impractical for a clinical application.

### ECG recording hardware

The results achieved in this work are based on 12-lead ECG signals. However, the hardware which was used to record the 12-lead ECGs is not MR safe and hence it cannot be used during MR imaging. Up to now, there is no MR safe 12-lead ECG system available on the market but a commercial 12-lead ECG system was made MR conditional previously [32,33]. With the results obtained in this work showing the superior signal quality of the precordial leads, such hardware could be promising for future applications – whether for gating in UHF CMR or for ECG-based diagnostics during MRI.

### Future work

The demixing matrix **W** was only estimated once for each dataset. Where this approach was successfully applied to nine datasets D_1_-D_9_ of healthy volunteers, problems could arise for patients with cardiac pathologies. For such cases, a recalculation of **W** could be required during runtime. This needs to be evaluated with additional datasets of volunteers or patients suffering from myocardial pathologies.

Due to the lack of MR-conditional 12-lead ECG hardware devices, contaminations caused by the switched gradient magnetic fields of the MR scanner have not been investigated. In this case, however, the demixing matrix **W** would be computed before playing the gradients. The resulting IC ${\hat{s}}_{\text{k,ECG}}$ is a linear combination of the original ECG leads. Based on these linear combinations, the gradient artefacts in ${\hat{s}}_{\text{k,ECG}}$ will still respect the linear time invariant assumption needed for the application of previously published methods for suppressing the gradient artefacts [21,34,35].

## Conclusion

Due to the MHD effect, the state-of-the-art VCG-based gating technique is prone to errors in 7 T CMR. To cope with this problem, an approach based on ICA was developed and applied to 12-lead ECG signals recorded inside a 7 T MR scanner. An IC ${\hat{s}}_{\text{k,ECG}}$ which was dominated by the R-peak was estimated by ICA. This IC was automatically identified and used for R-peak detection. Given the high R-peak detection quality within the test dataset (*Se* = 99.2%, +*P* = 99.1%), the proposed ICA-based technique outperforms the state-of-the-art VCG-based gating method (*Se* = 72.1%, +*P* = 56.4%).

Previous investigations revealed that the precordial leads of the ECG are less affected by the distortions caused by the MHD effect [22,36]. The importance of the precordial leads for the proposed method was demonstrated by applying ICA to several lead configurations (LC2) with different combinations of the limb and precordial leads and by using a single ECG lead for R-peak detection (method M1). This finding could be helpful for the development of improved MR-conditional ECG hardware with additional precordial leads or for further investigations regarding the placement of the ECG electrodes.

## Endnotes

^a^ Image source (Figure 1): https://upload.wikimedia.org/wikipedia/commons/f/f4/Wiggers_Diagram.svg

## Abbreviations

CMR: Cardiovascular magnetic resonance; ECG: Electrocardiogram; Ff: Feet first; Hf: Head first; ICA: Independent component analysis; MHD: Magnetohydrodynamic; MRI: Magnetic resonance imaging; PC: Principal component; UHF: Ultra high field; VCG: Vectorcardiogram.

## Competing interests

The authors declare that they have no competing interests.

## Authors’ contributions

The initial manuscript was read critically and edited by all authors. JWK, GDC and JO developed and evaluated the algorithms. JWK designed and conducted the measurements and implemented the algorithms. All authors read and approved the final manuscript.
